# Independent Journalism from a Dental Stand-Point

**Published:** 1898-12

**Authors:** George S. Allan

**Affiliations:** New York City


### International Dental Journal.

Vol. XIX. December, 1898. No. 12.

### Original Communications.[1]

### INDEPENDENT JOURNALISM FROM A DENTAL STAND-POINT.[2]

BY GEORGE S. ALLAN, A.B., D.D.S., NEW YORK CITY.

Mr. President and Members of the American Academy of Dental Science,—For years the profession of dentistry has been drifting, drifting, but, nevertheless, advancing, moving onward; now heading, in its haphazard voyage, for one port, now for another, without guidance, without direction; gaining knowledge and skill, but not knowing where to seek a permanent harbor; gathering, on its varied craft, a rich fruitage of possibilities for benefiting mankind, but, alas! not owning a reliable ship to place it in, nor having a captain to take command. No one questions that dentistry is a branch of the healing art, and a most important one, but dentists are recognized as doctors only in name. The dental diploma neither admits the possessor to membership in medical societies nor gives him the benefits and advantages which rightly belong to him as an integral factor in one of the most learned, and, from a humanitarian stand-point, most appreciated and honored of the professions. All recognize this fact, but only a few see clearly why it is so, or are in

[1] The editor and publishers are not responsible for the views of authors of papers published in this department, nor for any claim to novelty, or otherwise, that may be made by them. No papers will be received for this department that have appeared in any other journal published in the country.

[2] Read before the American Academy of Dental Science, May 4, 1898.

a position to point out the road to reform and to lead the way. If dentists have lost their birthright, they may be certain that the fault lies, not with their parents, but with inherent views and habits of the offspring. Man loves life, and this love of life and health sought a mate and found it. The mate was called Knowledge,—knowlege of the laws governing life and health, and of the means for regulating and controlling them. The child of this "love of life" and its mate, Knowledge, was called Medicine. But medicine does not call one part of the body essential and important, and another not so. It does recognize differences in degrees of importance and value, but at the same time clearly asserts that all are mutually related to and dependent on each other. The truth is, the teeth are all-important for health and comfort, and their good or bad condition makes for human happiness or the reverse, and the dentist in no way occupies a second position in the great domain of the healing art. Why, then, does he willingly take a back seat? What influences are at work to keep him from his birthright, and what can be done to overcome them?

Burns, who sang so sweetly on many themes,—alas, that he should have left the world so young in years!—saw clearly many things relating to human life, and one want of power he most beautifully and strongly presents to us in his memorable lines,—

"Oh, wad some power the giftie gie us
To see oursel's as others see us!
It wad frae monie a blunder free us,
And foolish notion."

There is much wisdom in this homely verse. Let us try to avail ourselves of it by putting ourselves outside of our daily life, and endeavoring to take a correct view of things as they are.

First, dentists do not practise their art *ex cathedra.* As a class they do not hold medical diplomas. There are many reasons for this, some good, some bad, but the parent profession is in no way responsible for any of them. At all times that profession has been ready and willing to extend to us a welcome, acknowledging the right and justice of our claims, but at the same time it has said that we were not entitled to special privileges. In other words, recognition could not be extended to any but those who hold the medical diploma, and to obtain that a special course of study and preparation was necessary. So soon as dentists put themselves in line with the other branches of medicine they would be received into the fold. No criticism can be made on this action, or lack of action, on the part of the medical profession. If they should open the doors to us without this formality, it would not be long before the oculist, the gynæcologist, and all other specialists would be claiming the same rights, and there would be an end to the unity of the profession. But there is no time to discuss this phase of the question. In good time the operation of natural laws will bring about its proper solution. It remains for us only to prepare the way, as far as possible, to remove obstacles, and trust to time and the normal growth of the profession on general principles to complete the work. Then we can and will take our right position naturally and easily. It is the part of wisdom, then, to order our lives and conduct in such a way that when the time comes we shall be ready for it.

To practise medicine two things are essential: first, the diploma, which means that the holder has taken the prescribed course of study and passed his examinations; second, that he shall live up to a special code of ethics; and these latter are based not on moral law only, but have added to them certain other principles, the meaning of which is that those who practise the healing art must order their lives and practice on different lines from those who engage in commerce and trade. These latter laws say that the physician occupies a different position in offering his services to suffering humanity from the merchant selling his goods. I do not for a moment suppose that the medical code of ethics implies any claim, on the part of the medical profession, that its members occupy any higher ground in the moral law than other workers do. The moral law is for all humanity. It governs and controls all alike. Therefore these additions to the moral law, which, after all, are the groundwork, the warp and the woof, of the whole code, are intended only for regulating the life and work of doctors on certain prescribed lines. They are the result of years of study and thought, they are fundamentally correct, they exert a most salutary and beneficial influence, and they are far more likely in the future to be strengthened and added to than to be dropped. The reason for this it is not at all necessary in this place to enter into. All of you are acquainted with the rules of the medical code, and their meaning and power for good. We have but to accept them as truisms.

In what respects do the dental codes of ethics differ from those of medicine? and—what is more to the purpose—to what extent does the profession enforce them? These are pertinent questions at this time.

It may be stated that dental codes do not differ in essentials from medical codes, but in their enforcement there is a wide difference. The former, to a great extent, are dead letters, and little or no attention is paid to them; and for this sad condition there is but one explanation,—the profession, as a whole, does not believe in them. Just the contrary condition holds with physicians as a class. As a whole, they do believe in codes, written or unwritten, and their rigid enforcement follows, as a matter of course, without question. The *esprit de corps* is alive and active in the one case, and nearly inanimate in the other. The tendency in the one case is to detach the profession entirely from trade influences, and to black-list without mercy those who hold to them. In the other it is to make use of such influences, if not openly, at least covertly, and to excuse those who employ them. Few of those who are posted and have given the subject any attention will question this broad statement. So it amounts to this: dentists are not doctors, first, because they do not hold the medical diploma, and, secondly, for the reason that they do not believe in or live up to the medical code of ethics. *In posse* they are doctors, *in esse* they are not; they occupy a sort of border-land, and so do not hold the respect of or confidence of either of their neighbors. The right hand of fellowship and comradeship is not extended to them by either party, and so, perforce, they have to, as the saying is, meet with themselves.

Some of our brothers seem to be happy in the idea that we are sufficient unto ourselves, and that we neither want to nor can be benefited by any alliance with medicine in general; that it is better to "go it alone," and so gain in self-respect and strength of character, power, and influence.

Well, I wish some of those who profess this belief would show us just where the line of separation between doctors and dentists can be placed, or give us a satisfactory definition,—the term of a specialty of medicine, one that would exclude dentists, and at the same time admit the hosts of other specialists that seek refuge under the paternal roof; or possibly they can tell us why, if it is better for us to live independent lives, it would not be equally advantageous for some of our professional relations to follow the same course, and why they do not endeavor to break the shackles that deprive them of a coveted liberty like unto that which makes us happy and prosperous. Are they blind, or only stupid? Or is there a real or fundamental difference between our callings? Is there an actual difference between the tissues and organs of the mouth and other parts of the body? One so radical that he who provides for the health and comfort of the dental organs and adjacent related parts must needs have a different title and position from his brothers?

If a good and sufficient answer cannot be given to these queries, then there is no reason why we should not seek our relations. If "blood is thicker than water in human affairs," so like views of life, its duties and responsibilities, should join together in an harmonious whole those who seek common ends.

Those who practise dentistry as an "art" must needs coin a new definition of the word if they wish to make it appear that they practise on lines different in any of its essentials, except in degree, from those who are disciples of the "healing art." The use of the term simply misleads. The desire is evidently to push to the front the æsthetic meaning of the word and give it undue prominence.

It would appear, then, that those who aim to broaden the lines of their work by merging their specialty in the general practice of medicine are but seeking that which is rightfully theirs already. In no sense of the word can it be said that they are trying to push in where they are not wanted, or that they are toadying to any set or class of men. They simply demand recognition of what they are,—nothing different, either better or worse. When they have obtained this, then they will be fully prepared to do their full share of all the work demanded of them, to accept their responsibilities, and give a full equivalent for all benefits derived.

If as much attention were given to the anatomy, physiology, and pathology of the teeth in the regular courses of medicine as is given to other parts and organs of the body, much benefit would accrue to both the general practitioner and to the specialist. The former would not make the many mistakes and blunders in diagnosis and treatment to which we dentists so frequently have our attention called. It is not an uncommon thing for the dentist to think, "If Dr. X. does not know any more about the other organs of the body than he does about the teeth, I am sorry for his patients." On the other hand, if the dentist were educated as a doctor, and his professional education founded on the principle that the teeth in no essential respect differ from the other organs of the body,—that they are living parts of a living whole,—he would not be less of a mechanic, but he would be more of a physician, and so every one concerned would be benefited, humanity most of all.

It is more than probable that the condition of affairs of which I have spoken is largely due to inherent causes, those that operate from within the profession and have their origin in the nature of the services rendered, which are largely mechanical, calling for special tools and appliances; these in turn force the dentist to be a mechanic, and cleverly and rightly encourage the inventive genius. The dentist who invents something new and valuable, not being held in control by the professional code, naturally seeks to profit by his labors and study, and is sorely tempted to take out a patent. As conditions are, I, for one, do not much blame him for the position he takes. We can hardly hope to see a rule that does not hold all in its grasp enforced from sentimental reasons only. Self-interest is all-powerful, and the ready excuse, "I am only doing what others do," is ever ready as a salve to the conscience. To the great credit of the profession, however, it can be safely said that it is steadily turning its face against the practice, and few self-respecting dentists to-day can be found who will either countenance the practice in others or make use of it themselves.

Of all the influences that have operated to the disadvantage of the profession and have tended to keep it away from the true professional standard, no one can be compared with that of the dental depots. These great establishments are organized and conducted on a strictly commercial and trade basis, and naturally and rightly conduct their business on laws and methods governing such establishments. They seek first of all their own good, look to their own interests, and care nothing for the advancement of the profession except in such ways as will add to the volume of their sales and their annual credit balance. No fault can be found with this fact, either as to its truth or the principles involved. The proprietors of these depots are in the business to make money, and it is folly to suppose for an instant that any other motive to any great extent influences them, or that they could be expected to part with any trade advantage for the sake of the professional *esprit de corps* among their customers. I see no reason to question their right to manage their business in their own way so long as they do not offend the general moral law; and, as a whole, I think they are managed honorably and justly, and aim to give, as they do give, a fair return for the cash received over the counter. Those I have known—the best—have been men of high character, and trustworthy in all business dealings. Still, I do not hesitate to say that the profession has lost more in prestige and power than it has gained in all other ways by the too intimate relations that have existed heretofore between it and these concerns. This relation has been a curious one, almost unique, for I doubt whether its like can be paralleled elsewhere. The depot has been an ever-present power, more so in the past than in the present, and the profession has taken kindly to its presence, guidance, and help in its various gatherings and conventions; has sought the depot's aid and taken its cash on many occasions when clouds seemed to be gathering and the future looked full of trouble. It is foolish to ask what were the motives of the depot managers on these occasions. It is just possible that they placed their money where they thought it would do the most good; and it is possible, for those who care to think so, that they were governed by high and generous motives. What was the opinion of the greatest among them, and the one who profited the most by his connection, may be judged by his indignant rejoinder to a question I put to him; asking me, "Where would the dental profession be to-day if it had not been for this great establishment?" Evidently he had the impression that if it had not been for the care, attention, and devotion of the foster-mother, the great establishment of which he was the head, the profession would still be in the pangs and dangers of parturition. I well remember the strong inclination I had to tell him that it was just possible that the care and attention the profession had received had not been altogether in the right direction; that it had been led into unnatural and erroneous ways, and that a good bit of manly, healthy vigor had been lost by the over-zealous and not altogether disinterested care bestowed.

The trade influence has kept alive and encouraged the whole system of patents and analogous methods, which are entirely anti-professional, if not absolutely ruinous to all the instincts and traditions and special laws of such a life; and, as if this were not enough, the whole system has been of a character which, while encouraging the individual, has defrauded the profession of such benefits as would follow were competition open and patents not allowed.

To the individual who has hit on a new device or principle, and has no scruples to benefit thereby, the manufacturer offers the customary royalty, gives him all the credit for his invention, pats him on the back, and tells him that he has a right to claim that which is justly his due as a result of his brain-work and study. To the unfortunate, who has some professional pride and feeling, he offers another bait,—one more suitable to his refined nature; it is somewhat like this, "Your views are quite right, and I respect your principles, but do you not see that in case the manufacturer is not protected, he cannot afford to give his time and capital to turning out goods when his competitors can at any time step in and compel him to share the profits thereon by manufacturing them themselves? It will require special tools and skilled labor to make a finished article, and these must be paid for, and if these are not given your invention will fall flat and you will get neither cash nor reputation. Now, if you will take out a patent on this valuable appliance and give me the sole right of manufacture, I will see to it that the goods are well and properly made according to your directions, and will charge only a fair manufacturer's profit, minus the royalty that would otherwise have gone to you." This bait has caught many well-meaning brothers, who have repented at leisure and in sorrow when they have found that they bartered their heritage of principles for a mess of pottage, and that their protestations of innocence and of good intentions were only, at best, half believed, and who with clearer vision have been made to see that open competition would have compelled a manufacturer to do his best to hold the market; that the invention, if valuable, would have pushed its own way, and that many sources of supply would have added to his credit and reputation by making it more widely known and appreciated.

A valid patent gives the holder thereof the sole right to manufacture at such times and in such quantities as may suit his convenience or expected gain. The working of this law has resulted in depriving dentists of the aid, advantage, and benefit of many valuable and helpful tools and methods of practice. For, as often happens, one invention is likely to interfere with another which accomplishes the same purpose in a different and perhaps simpler way. Of course, the manufacturer who has capital invested in one invention does not want to be interfered with by a rival, and so buys up the new invention and locks it up until such time as he can safely and prudently allow it to see the light. In this way the profession, as a whole, has suffered greatly, and out of all proportion to any benefits derived.

I have wandered too far from the real purpose of this paper, and now ask your attention to consider with me the true meaning and influence of the terms "independent" and "trade" as applied to journalism.

It is rather a startling assertion to make, still I make it unhesitatingly and without a doubt as to the correctness of my position, that the depots have captured our literature, and have been and are using it solely for mercenary trade purposes, and that in all doubtful cases or questions, the good of the trade takes precedence of the good of the profession. Now the literature of any profession or calling in life ought to be kept clean and pure and free from admixture with deteriorating influences. It should neither directly nor indirectly give aid or support to those who differ from it in the essentials of its life and being. Least of all should it be allowed to become the main support of any manufacturing establishment in pushing its wares on the market. The holding of these views does not in any way militate against an independent journal devoting a certain number of its pages to advertisements, accepting those that properly belong to its purposes,—those that will add to its value and help its income. But the literary department should control and direct the advertising department, and that is a vastly different state of affairs from that in which the trade department controls and manages the literary department, and uses it simply as a means of pushing its advertisements where they will do the most good. The art of advertising is important and valuable just in proportion as it can be made to attract the eye and reach the proper class of readers, and business men devote large sums of money for these purposes and employ special talent to make their announcements attractive. The dental depots save the greater part of this outlay, the dentists themselves kindly furnishing in most cases the special talent, gratuitously at that, and then paying for the "advertisements" in cash subscriptions for the magazine. No other class of business men are so fortunately situated as these publishers. They get much for little and have the laugh all to themselves! The subscriptions pay the bills and the advertisements cost them nothing or next to nothing. And yet you will find dentists and classes of dentists and societies who support this very pretty arrangement and say it is all right and "so handy." The depots appreciate all their advantages, but do not change their colors; they are the same old tradesmen,—just the same, and the tradesmen's codes of ethics, and those only, regulate their business life.

But this is not all. The former editor of one of our leading journals openly claims that his journal had made the reputations of many of the prominent members of our profession. He believes what he says, and, as the men were worthy of the honors conferred, feels naturally proud of his work. I do not question the correctness of his statement; but when I first heard it, the thought came into my mind, Cannot the same power that makes one man's reputation destroy another's, or keep him from obtaining his just dues? and is it right or proper to put so great a power for good or evil in the hands of those not guided and controlled by the same code of ethics that we live under? Think of it. Tell me, if you can, honestly, whether such power should live outside our ranks?

Much stress has been laid on the claim made by the leading trade journals that the editors are free to manage their department as they think best. True, indeed, to a great extent, but in all these cases the editor has no control whatever over the advertising pages, —in fact, has nothing to do with them. So these journals at the best are only half professional, and the other half trade; and when you come to think of it, they are literally all trade; for the sweeter, the more pleasant, and the more digestible the editor makes his pages, the more push the whole will have in the right direction; and that is his special business. How blind must a man be who cannot see through so big a hole! No wonder all dental depots want a journal, and want it badly; for their success and life depend on it. Years ago, one of the heads of a depot, now out of existence, said to me, "I want an organ and must have one, or I cannot compete with these big fellows." Shortly after he started one,—he had to,— but success did not go his way, and he gave up the fight.

The plea made by those who support the trade journals is that few or none of the dental societies can afford to publish their own proceedings; they have not the means, and therefore it is a matter of necessity to give them to the trade journals, and a few societies —to their shame be it said—accept pay for them. Well, it does look, as the saying is, rather "off color" for a reputable society to sell its proceedings. But think a little: in what respect, but one of degree, does the society that sells its proceedings for cash differ from the one that gives its proceedings to the same journals? In the one case the society gets its proceedings published free of cost; in the other, it obtains the same advantages plus so much cash. In both cases it amounts to so much cash in pocket, and that is all. No, it is not all. There are some things that honorable men do not sell, least of all are they willing to sell their principles.

> "Who steals my purse steals trash;
> But he that filches from me my good name
> Robs me of that which not enriches him
> And makes me poor indeed."

Honorable men will not part with any of their self-respect, not for cash, at all events.

Taking the most charitable view of the case possible, the society which gives its proceedings to a trade journal puts itself under obligations to that journal. Well, some do not relish the thought of accepting such obligations, and do not rest content when they have to accept them. They become restless and discontented, and make every effort to get rid of them, for they find themselves in a false position. There is an old saying that he who accepts a favor must take a kick before he can get rid of it. It seems to me that our profession has got its kick, but it has not got rid of the obligation. It may serve our purpose to make a direct comparison between the two classes of dental journals.

The trade journal is in all essentials the organ of some dental depot or manufacturing concern. It is made for profit, published purely as a matter of business, and as a whole is simply an advertising medium connecting the dental depot and its customers. For convenience and policy's sake it is divided into two parts, the literary and the advertising. The advertisements alone would have no push or power, and would in a great measure fall flat, and find a quick burial in the waste-paper basket. Even if glanced at, or partly read, they would quickly be forgotten or mislaid. They must be kept alive, in evidence, ready for reference, and where they can be seen, or they will fail. So the literary department is most wisely attached to rescue the advertisements from their inherent weakness, to give them life, character, influence, and the power of self-preservation. The dentist takes his journal in order to keep himself posted in matters relating to his calling. He feels that he must keep abreast of the times, and prides himself on his ability to give his patients the full benefit of the labors and thoughts of his fellow-workers; and for their value in this way the papers are kept for reference and study, and they are kept in whole and not in part. Nominally, the literary part of the journal is of first importance, but in reality the advertising half holds that position, and the journal would have no meaning to the publishers if this relationship did not exist. Now, I, for one, hold that it is a palpable infraction of the moral law for a trade journal to print on its title page "A monthly record of dental science, devoted to the interests of the profession." More honest and correct would it be if made to read, "A monthly record of dental science, published in the interest and for the benefit of the X Y Z dental depot." I strenuously object to seeing the professional flag floating over a trade fortress. It may look pretty, but it is sadly out of place. It is deceptive and hides a masked battery.

The independent journal stands for quite a different thing. It stands for the profession and the profession only. It means the absolute control of the advertising as well as the literary matter, and means that the latter should in fact as well as in name be the essential part of its existence. In no way whatever should it be published for profit, except inasmuch as the profession may reap the whole profit thereof. Every dollar received in excess of the cost of manufacture should be turned into the common fund, and be used solely for improving the journal or as a safety fund. It should not be allowed to contribute to the profit or advancement of any individual or class. The journal should float its own colors and fight for them. A high professional standard should be insisted on, and advocated in all proper ways, by example as well as precept.

A journal or journals—and they should be many—so conducted and supported by the profession ought to have and would have a marked influence for good, and go far towards relieving the profession from the trade-influence incubus it now labors under.

In 1890—I speak from memory—I had the satisfaction of having a contribution on this subject refused by the *Dental Cosmos.* The reasons given were trade reasons only, and had nothing to do with the real merit of the article, which, indeed, was admitted. Before sending it, it was submitted to several of our leading men and by them fully endorsed in all respects. This is not an isolated case.

It is curious how habit and custom confuse thought and blunt the moral perceptions. For so long a time have dentists either given away their literature, or marketed it to the highest bidder, that they have ceased to consider the highest questions involved, or the obligations they rest under to the profession as a whole.

The strong societies ought to meet the issue fairly and squarely, set the fashion, and pay their way, including the salary of the editor, and then the weaker ones will gradually fall into line to the extent of their ability; all will gain thereby in self-respect and in influence. The editor of a trade journal is employed by the depot managers—his salary is paid by them—and is in all essentials their servant. Can he serve two masters? The greatest of all authorities says, *No!* No matter what his impulses or ambitions may be, he is not, and in fact cannot be, a free man.

As a rule, mankind values most that which costs the most. If dental literature is valuable to the depot managers as a means for selling their goods, it needs must have as great or a greater value to the profession for disseminating knowledge and principles. It is most reasonable, then, for the societies to pay a little more and keep the reins in their own hands,—in other words, to pay a journal owned and supported by the profession a fair sum for publishing their articles. Even were they to gain in no other way by so doing, they would gain much in self-respect, and that in this instance amounts to a great deal. But a greater good lies hidden under this little matter of dollars and cents. I have too great faith in my brothers to believe that when they once fairly grasp the principles involved they will longer submit to their unworthy thraldom.

In what I have said there may be some strong or even harsh expressions. If they so appear to any of you, kindly overlook them, and credit me only with a strong and earnest desire to advance our common interests, ambitions, and hopes on correct lines.

I know of many good men who hold contrary views from those which I have expressed, and I respect them and their views, for they are as honest as I am, and equally desirous of advancing the good cause. I am sorry that this difference of opinion exists. In the end the right and the best will prevail.

Rossini, in his famous opera, "The Barber of Seville," written in the early part of this century, puts into the mouth of Figaro, where he boasts of his skill, "I am barber, surgeon, and jack of all trades, and my pockets are never empty," and later on he says he knows all about the people, as he is barber, hair-dresser, surgeon, and major-domo of the household. At this period the surgeon and the dentist occupied similar positions; neither commanded the respect of the people. They were tinkers, and tinkers only. As time went on, the surgeons, step by step, advanced, and, joining hands with the physician, pushed their department of medicine into great prominence, and to-day no class of men can claim a prouder position than they. Times have indeed changed with the surgeon, but the calling of the dentist still lags behind.

If, now, you will turn to your copy of the Century Dictionary, the last edition of which has but recently been published, you will find that a dentist is "one whose profession it is to clean and extract teeth, repair them when diseased, and replace them, when necessary, by artificial ones; one who practises dental surgery and mechanical dentistry."

How do you like the pen-picture which this great authority places before your eyes? The first definition given in a dictionary is generally the main one, the one commonly accepted as correct. The definition quoted is not quite up to date so far as the opinions of some of you are concerned; but, alas! we must take the position assigned to us by the combined verdict of the masses, and, in the main, I believe the masses judge rightly, for their judgment is founded on that estimate which any man or set of men place on themselves and live up to.

In the early dawn of the twentieth century I hope to see the definition read something in this way: "Dentistry is a specialty in medicine, dealing with the oral cavity, with especial reference to the medical and surgical treatment of the teeth." If that good day is to come, as soon I hope and believe it will, then we must meet together with a common purpose and work for what we believe. The fountain-heads of instruction, our colleges, societies, and journals, must be made to push the thought and principle both by precept and example. The student must grow up with the thought that he is in fact a doctor of medicine,—not half doctor and half tinker. The time for push and work is at hand, and all lovers of their profession should take part and not rest till the prize is won.

> "There is a tide in the affairs of men
> Which, taken at the flood, leads on to fortune;
> Omitted, all the voyage of their life
> Is bound in shallows and in miseries."

Gentlemen, I believe the tide is now in our favor, and that the time for work is at hand. The subject, as has been well said, is in the air, and it needs but a strong and united effort to mount the crest of the wave that will lead us on to fortune. The faith that is in us must be fought for earnestly, hopefully, and manfully, and the truth will surely prevail.

